# Deploying Implementation Strategies to Facilitate Professionals’ Use of the Home Care Frailty Scale

**DOI:** 10.1093/geroni/igab046.721

**Published:** 2021-12-17

**Authors:** Lisa Juckett, Haley Oliver, Leah Bunck, Crystal Kurzen, Andrea Devier, Fannisha Page

**Affiliations:** 1 The Ohio State University, Columbus, Ohio, United States; 2 LifeCare Alliance, Columbus, Ohio, United States

## Abstract

Home- and community-based service (HCBS) organizations play an instrumental role in maximizing the independence of older adults, ages 60 and over. HCBS clients typically have multiple health complications, placing them at great risk of frailty—a complex condition associated with health decline and institutionalization. However, despite their frequent contact with older adults, HCBS professionals are not required to assess the frailty levels of their clients, creating a missed opportunity to monitor the needs of this at-risk population. The purpose of this quality improvement study was to test a package of five implementation strategies designed to support HCBS professionals’ use of the evidence-based Home Care Frailty Scale (i.e., Frailty Scale) with all new clients at one large HCBS organization. Implementation strategies included (a) selecting one professional to serve as the organization’s Frailty Scale “champion,” (b) holding three training sessions with 25 HCBS professionals, (c) modifying client charts to allow professionals to document Frailty Scale results, (d) pilot testing the Frailty Scale with a small group of clients, and (e) completing monthly chart audits to monitor rates of Frailty Scale implementation. During the first three months of Frailty Scale use, HCBS professionals administered the Frailty Scale to 414 out of 467 eligible clients (88.6%). For Month 1, 87.4% of eligible clients were administered the Frailty Scale, followed by 90.8% in Month 2, and 85.6% in Month 3. This quality improvement study suggests that a multifaceted package of implementation strategies can support professionals’ use of an evidence-based frailty instrument in the HCBS setting.

